# Examining the long-term cognitive effects of exposure to the Canterbury earthquakes in a resilient cohort

**DOI:** 10.1192/bjo.2022.512

**Published:** 2022-06-15

**Authors:** Caroline Bell, Will Moot, Richard Porter, Chris Frampton, Virginia Mcintosh, Melissa Purnell, Rebekah Smith, Katie Douglas

**Affiliations:** Department of Psychological Medicine, University of Otago, New Zealand; Department of Psychological Medicine, University of Otago, New Zealand; Department of Psychological Medicine, University of Otago, New Zealand; Department of Psychological Medicine, University of Otago, New Zealand; Department of Psychology, University of Canterbury, New Zealand; Department of Psychological Medicine, University of Otago, New Zealand; Canterbury District Health Board, New Zealand; Department of Psychological Medicine, University of Otago, New Zealand

**Keywords:** Trauma-exposed, resilience, earthquake, neuropsychological function, facial expressions

## Abstract

**Background:**

Although most people do not develop mental health disorders after exposure to traumatic events, they may experience subtle changes in cognitive functioning. We previously reported that 2–3 years after the Canterbury earthquake sequence, a group of trauma-exposed people, who identified as resilient, performed less well on tests of spatial memory, had increased accuracy identifying facial emotions and misclassified neutral facial expressions to threat-related emotions, compared with non-exposed controls.

**Aims:**

The current study aimed to examine the long-term cognitive effects of exposure to the earthquakes in this resilient group, compared with a matched non-exposed control group.

**Method:**

At 8–9 years after the Canterbury earthquake sequence, 57 earthquake-exposed resilient (69% female, mean age 56.8 years) and 60 non-exposed individuals (63% female, mean age 55.7 years) completed a cognitive testing battery that assessed verbal and visuospatial learning and memory, executive functioning, psychomotor speed, sustained attention and social cognition.

**Results:**

With the exception of a measure of working memory (Digit Span Forward), no significant differences were found in performance between the earthquake-exposed resilient and non-exposed groups on the cognitive tasks. Examination of changes in cognitive functioning over time in a subset (55%) of the original earthquake-exposed resilient group found improvement in visuospatial performance and slowing of reaction times to negative emotions.

**Conclusions:**

These findings offer preliminary evidence to suggest that changes in cognitive functioning and emotion processing in earthquake-exposed resilient people may be state-dependent and related to exposure to continued threat in the environment, which improves when the threat resolves.

Over 2010–2011, Canterbury experienced four major earthquakes (moment magnitude scale >6.0) and thousands of aftershocks, resulting in major property and infrastructure damage throughout the city, 185 deaths and thousands of injuries.^1^ A large number of people were exposed to the earthquakes, creating a unique opportunity to examine the cognitive and emotional effects of trauma exposure.

Epidemiological studies have shown that exposure to potentially traumatic events is common, with 70–80% of people experiencing one or more such events over their lifetime.^2^ Although there is increased risk of developing a mental health disorder after such exposure,^3^ this occurs in only a minority.^4^ Most people follow a trajectory of recovery or resilience, with relatively short-lived distress that settles over time.^5^ Although extensive literature reports on the impact of trauma on brain functioning in post-traumatic stress disorder (PTSD), an increasing number of studies have examined outcomes in trauma-exposed but resilient participants, who constitute most of the population. These studies have provided insights into the impact of exposure to trauma on neurocircuitry, cognitive functioning and resilient responses.

## Brain functioning and resilience

Neuroimaging studies comparing groups with and without PTSD after trauma exposure have shown greater prefrontal–amygdala activation or connectivity in resilient (without PTSD) groups,^6^ which, it is hypothesised, reflects greater top-down regulation of emotion and a resilient response.^7^ Studies specifically examining the effects of exposure in individuals without PTSD have reported long-term changes, with greater grey matter density in the prefrontal–limbic systems compared with non-exposed controls.^8^

Studies of cognitive functioning in PTSD report that differences are significantly less when the comparison group includes trauma-exposed rather than non-trauma-exposed controls.^9,10^ This suggests that trauma itself may have detrimental effects on cognitive performance not only in people with PTSD, but also in exposed but psychiatrically well people. This would provide support for reports in the general population of ‘earthquake brain’ or ‘flood-brain’ after trauma exposure.^11^ Studies specifically addressing this issue have had inconsistent findings. For example, Stein et al. showed that women exposed to intimate partner violence, regardless of PTSD status, had impairment in tasks of attention, working memory, and response inhibition, compared with non-exposed controls.^12^ After exposure to the Canterbury earthquake sequence, no difference was found in performance on cognitive tasks between individuals with PTSD and resilient trauma-exposed individuals. However, both of these earthquake-exposed groups had poorer performance on a test of visuospatial learning and memory compared with non-exposed control groups.^13^ In individuals exposed to urban violence, Flaks et al found no differences in attention and executive functioning between an exposed but psychiatrically well group and a non-exposed control group.^14^ These inconsistent findings may be explained by factors such as age at the time of exposure, type (e.g. natural disaster or sexual assault) and duration of trauma (e.g. single incident such as a motor vehicle accident or chronic repeated events such as intimate partner violence).

Facial emotion processing is a specific aspect of cognitive functioning that has received less attention in relation to trauma exposure. A systematic review of studies of social cognition in PTSD reported mixed findings in the processing of threatening expressions (anger, fear, sadness).^15^ Studies in people with PTSD found decreased accuracy and sensitivity in interpreting facial expressions of fear, sadness,^16,17^ and anger,^16^ compared with combat-exposed controls. Studies, including our own work following the Canterbury earthquakes, have found that when compared with non-exposed controls, exposed individuals (including those without PTSD) had increased sensitivity for cues of potential threats, including emotional facial expressions, which could help interpret potentially harmful situations rapidly and may therefore, in the short term, be a useful adaptive response.^18–20^

We previously assessed cognitive functioning and emotion processing approximately 2–3 years after the Canterbury earthquake sequence in 89 individuals who were exposed to these events but did not develop psychological difficulties, and identified as resilient.^13,18^ Similar to those with PTSD, resilient individuals showed clinically significant impairment in visuospatial learning and memory and facial emotion processing compared with a non-exposed group who had completed cognitive testing in other studies before the earthquakes. These findings highlight the importance of understanding cognitive functioning in resilient groups in addition to those with mental health disorders. This may be particularly important after disasters because of the population-wide effects and a resilient response being the most prevalent. If there are persisting impairments in threat sensitivity and cognitive functioning, this may affect people's productivity and could potentially be a target for intervention.

The aim of the current study was to focus on the earthquake-exposed resilient group from our previous work to examine the longer-term cognitive effects of the Canterbury earthquake sequence (approximately 8–9 years after exposure) in a context of reduced ongoing seismic threat (in contrast to the ongoing risk of further earthquakes at the time of the original study). The design was improved from the original study by recruiting a matched non-earthquake exposed control group and expanding the cognitive testing battery. In accord with our previous findings, the hypothesis was that participants in the earthquake-exposed resilient group, compared with non-exposed controls, would perform less well on tests of spatial memory, have increased accuracy for the identification of all facial emotions and exhibit a bias in the misclassification of neutral facial expressions to threat-related emotions.

## Method

### Participants

#### Earthquake-exposed resilient participants

This group comprised Canterbury residents who had self-identified as resilient, i.e., coping well, despite moderate-to-high exposure to earthquake-related events (such as physical injury or illness, death of a loved one, witnessing falling buildings, seeing bodies, property loss, income loss or problems with housing caused by earthquake-related events). They were recruited in response to articles, opinion pieces and community notices in local newspapers, and via word of mouth over the course of 13 months, from January 2013 to February 2014. They all had a face-to-face assessment and completed diagnostic and self-report questionnaires, to confirm that they had no earthquake-related psychiatric diagnoses and had not received any earthquake-related counselling. In this current study, this group were retested between July 2018 and March 2020 (approximately 8–9 years after the Canterbury earthquakes in 2010 to 2011). They were recruited from the 101 people who participated in the original studies approximately 2–3 after the Canterbury earthquakes and had consented to be contacted for future research.^13,18^ Reasons for exclusion from both the original and current study were current alcohol dependence, lifetime psychotic or bipolar disorder, comorbid neurological or medical conditions, pregnancy, previous serious head injury or taking medications likely to interfere with cognitive testing. All participants were fluent in English.

#### Non-earthquake-exposed controls

Non-earthquake-exposed control participants, recruited through advertising (using similar methods) between September 2019 and June 2021 in Dunedin, New Zealand (approximately 300 km south of Canterbury), had not been exposed to the earthquakes and had not lived in Canterbury since the earthquakes. Control participants were subject to the same exclusion criteria as earthquake-exposed participants. They were matched with the earthquake-exposed resilient group for age, gender and years of education.

### Procedure

Assessments were conducted by research assistants at university research units in Christchurch (earthquake-exposed resilient participants) and Dunedin (non-exposed controls). Each assessment was part of a battery of tests (total duration approximately 180 min), which included diagnostic and psychometric measures, cognitive assessment and a narrative of earthquake experiences (earthquake-exposed group only, reported separately). Research assistants received training in administration of cognitive testing by a clinical psychologist (K.D.).

#### Cognitive testing

Cognitive testing included a battery of tasks designed to test visuospatial learning and memory, verbal learning and memory, executive functioning, psychomotor speed, sustained attention and social cognition. Pencil-and-paper tasks were administered according to standardised instructions, and computerised tasks according to corresponding manual protocols (E-Prime 2.0 software (Psychology Software Tools, Inc, Sharpsburg PA, USA; see https://pstnet.com/products/e-prime/) and CogState software (CogState Inc, Fitzroy, Melbourne, Australia; see https://www.cogstate.com/academic-research/)) on a PC laptop. The following tasks were administered.

##### Verbal learning and memory

For the Rey Auditory-Verbal Learning Task (RAVLT),^21^ participants recalled words from a list presented five times (trials 1–5), after a distractor list (trial 6) and after 20 min (trial 7, delay). Number of words recalled in each of the trials from 1 to 7 was recorded, as well as distractor list recall and total learning (sum of trials 1–5).

##### Visuospatial learning and memory

For the Groton Maze Learning Test (GMLT; CogState), participants navigated a 28-step hidden pathway within a 10-by-10 grid of squares on a computer screen. The process was repeated for four successive learning trials (total of five learning trials) and a delay trial after 20 min. The number of errors for each trial, and over all learning trials, was recorded.

##### Executive functioning

For the Digit Span Forward and Backward test, participants were read a sequence of numbers and asked to repeat the sequence in order (forward) or in reverse order (backward). Total score (number of sequences correctly recalled) and span length (highest number of digits recalled) were recorded for forward and backward, as well as a total score (forward + backward score).

For the Controlled Oral Word Association Test, a task of verbal fluency, participants generated words starting with a particular letter (C-F-L), for a period of 90 s. The number of words generated for each letter was recorded and summed across the three letters, giving a total score.

The Delis–Kaplan Executive Function System – Fluency Battery^22^ involved two measures of verbal fluency: category fluency, which required participants to generate as many words as possible from designated semantic categories (animals, boys names), and category switching, in which participants alternated between generating words from two different categories (fruit, furniture). Total words generated in both conditions, and total correct switches in the latter condition, were recorded.

##### Psychomotor speed

The Timed Chase Test (TCT, CogState) served as a control task for the GMLT and involved participants chasing a moving tile around a grid of squares on a computer screen for 30 s. Number of correct moves per second was recorded.

The Digit Symbol Coding Test (DSCT) assessed participants’ ability to correctly draw symbols that corresponded with specific digits under time pressure (90 s). Number of correct responses was recorded.

##### Sustained attention

In the computerised Vigil Continuous Performance Test,^23^ single randomised letters were presented sequentially on a computer screen for 85 ms, with a 900 ms inter-stimulus interval. Letters were presented in white on a black background. Participants responded when they viewed target ‘K’, only when cued by an earlier ‘A’ stimulus (‘AK’ target sequence). Over the course of 480 stimuli, 100 target sequences are presented in 8 min. These targets were pseudo-randomised, so that 25 target sequences were presented in four blocks (with no breaks between blocks). The number of omissions and commissions were recorded.

##### Social cognition

The Reading the Mind in the Eyes Test (RMET)^24^ is a 36-item task in which participants indicated which emotion best matched the mental state that different eye images were displaying. The task used a pen-and-paper format with black and white images. Forced-choice responding was used, with participants choosing one of four adjectives for each item (e.g. jealous, hateful). There was no time limit to complete the task; however, participants were asked to answer as quickly and accurately as possible. A total score out of 36 was calculated, with higher scores indicating more accurate performance.

##### Facial expression emotion recognition test

For the Facial Expression Recognition (FER) Test, participants completed a modified version of the FER task^25^ presented on a computer, using E-Prime software (E-Prime 2.0). Faces displaying five basic emotions (happy, sad, angry, fearful and disgusted) were randomly presented on a screen for 500 ms, followed immediately by a blank screen. Faces had been morphed into varying intensities of each emotion from 50 to 100%, in 10% steps. Neutral facial expressions (0% emotion) were also presented. Participants were instructed to identify the emotions displayed in a forced-choice format as quickly and accurately as possible. Reaction times, recognition accuracy and neutral misinterpretation bias (percentage of neutral expressions misclassified as an emotion) were recorded (for more detailed description, see Douglas and Porter^26^).

The RAVLT, GMLT, TCT and DSCT were used in the original study 2–3 years after the start of the earthquake sequence. To prevent practice effects in the earthquake-exposed resilient group, alternative versions were used in the current study.

#### Diagnostic and psychometric measures

All participants had a diagnostic interview to screen for Axis I mental disorders (Mini-International Neuropsychiatric Interview; MINI), completed the National Adult Reading Test (NART) and the following self-report psychometric measures.

##### PTSD Checklist-Specific

The PTSD Checklist-Specific^27^ assesses 17 current (past month) symptoms of PTSD in relation to the Canterbury earthquakes (completed only by the earthquake-exposed group). Total symptom scores range from 17 to 85, with scores >44 being in the clinical range.

##### Depression, Anxiety and Stress Scale

The Depression, Anxiety and Stress Scale (DASS-21)^28^ assesses 21 symptoms (seven items for depression, anxiety and stress) over the past week. Subscale scores of >14 for depression, >10 for anxiety and >19 for stress are defined as moderate-extreme.

##### Dissociative Experiences Scale

The Dissociative Experiences Scale (DES)^29^ assesses amount of time participant experiences 28 symptoms of dissociation, with higher scores indicating higher dissociation.

##### Posttraumatic Growth Inventory

The Posttraumatic Growth Inventory^30^ assesses 21 positive outcomes from the earthquakes (earthquake-exposed resilient group only). Scores are summed and range from 0 to 105, with higher scores reflecting greater post-traumatic growth.

##### Connor–Davidson Resilience Scale

The Connor–Davidson Resilience Scale (CDRS)^31^ assesses 25 resilience items over the past month. Scores are summed and range from 0 to 100, with higher scores reflecting greater resilience.

##### Social Adjustment Scale

The Social Adjustment Scale (SAS)^32^ assesses 45 items of social functioning over the past 2 weeks. Scores are summed and divided by the number of items, and range from 1 to 5.

##### Traumatic Exposure Severity Scale

The Traumatic Exposure Severity Scale^33^ assesses the occurrence of 39 earthquake-related stressors and the distress experienced in relation to these (earthquake-exposed resilient group only).The number of exposures and distress scores are summed.

##### Life Events Scale

The Life Events Scale (LES) was adapted from the Crisis in Family Systems–Revised Questionnaire,^34^ and assesses 66 stressful life events (not specifically related to earthquake exposure) in the previous 5 years and the past 6 months. Number of exposures and distress scores are summed.

##### Childhood Trauma Questionnaire

The Childhood Trauma Questionnaire (CTQ)^35^ retrospectively measures childhood abuse and neglect, rating frequencies of experiences from 1 to 5. Five subscale (emotional abuse, physical abuse, sexual abuse, emotional neglect and physical neglect) and total scores are produced from summing scores.

##### State-Trait Anxiety Inventory

The State-Trait Anxiety Inventory (STAI) assesses state (current) and trait (lifetime) symptoms of anxiety, with higher scores indicating greater anxiety.

### Ethics

The authors assert that all procedures contributing to this work comply with the ethical standards of the relevant national and institutional committees on human experimentation and with the Helsinki Declaration of 1975, as revised in 2008. All procedures involving human patients were approved by the University of Otago, New Zealand Human Ethics Committee (approval number H18/042). All participants provided informed written consent.

### Statistical analysis

Statistical analyses were conducted with the Statistical Package for Social Sciences version 26 for Windows. Demographic and clinical data were summarised with standard descriptive statistics, including means, s.d., ranges, frequencies and percentages, as appropriate. Comparison of demographic and clinical variables between the earthquake-exposed and non-exposed controls used ANOVA and chi-squared tests. Comparisons of cognitive data were conducted with ANCOVA, with group as the between-participant factor and age, gender, years of education, anxiety from STAI-state and depression from DASS-21 depression subscale score as covariates to adjust for clinical features known to affect cognitive functioning. Ethnicity, CTQ score and NART score were also used as covariates to adjust for differences between groups. Where tasks had more than one level, an additional within-participants factor of level was added and analysed by repeated measures ANOVA, within a general linear mixed model. A supplementary analysis was completed of performance at 2–3 years (time point 1) and 8–9 years after the earthquakes (time point 2) in the resilient earthquake-exposed controls who had completed cognitive testing at the two time points, using repeated measures ANCOVA, but with an additional within-participants factor of time (time point 1 versus time point 2). A two-tailed *P*<0.05 was taken to indicate statistical significance.

## Results

From the 101 earthquake-exposed resilient participants identified in the original studies, 59 participated in the current study (15 unable to be contacted, seven not interested, four too busy, four unwell, four no longer in Canterbury and eight were members of the University department who, at the time of the current study, had considerable knowledge of the study design and testing). Two participants were excluded because of alcohol dependence when assessed (see Supplementary Figure 1 available at https://doi.org/10.1192/bjo.2022.512). The only statistically significant difference in psychometric and cognitive tests at time point 1, between those who participated in the current study and those who did not, was higher alcohol use before and after the earthquakes, and greater accuracy for neutral expressions from the FER, in the group who did not participate in the current study. Sixty non-exposed controls were recruited..

Table 1 shows that although groups were well-matched on gender, age and years of education, the earthquake-exposed resilient group scored significantly higher on the NART and included a greater proportion of people of New Zealand European origin. Three participants (5%) in the earthquake-exposed resilient group had a current diagnosis of generalised anxiety disorder. Both groups reported functioning well (from the SAS), had negligible rates of mental disorder (from the MINI) and low use of antidepressants. Both groups had low scores on measures of depression (from the DASS-21), anxiety (from the anxiety subscale of the DASS-21 and STAI) and dissociative experiences (from the DES), and high scores on measures of resilience (from the CDRS). Compared with the non-exposed group, the earthquake-exposed resilient group rated significantly higher stress (from the DASS-21 stress subscale), and reported exposure to a greater number of stressful life events and greater difficulty from these over the past 5 years (from the LES). The non-exposed control group scored significantly higher than the earthquake-exposed resilient group on total childhood trauma (from the CTQ emotional abuse and emotional neglect subscales), although all scores were in the categories of none or low abuse.

**Table 1:** Comparison of demographic and clinical variables between earthquake-exposed and non-exposed groups

	Earthquake-exposed resilient group (*N* = 57)	Non-exposed control group *(N* = 60)
Gender, % (*n*)	69% (41)	63% (38)
Age, years, mean (s.d.)	56.8 (11.4)	55.7 (11.5)
Years of secondary/tertiary education, mean (s.d.)	17.1 (4.1)	16.9 (3.5)
National Adult Reading Test, mean (s.d.)	116.0 (6.5)	111.5 (7.5)**
Ethnicity (New Zealand European), % (*n*)	89 (50)	71 (42)*
Current diagnosis on the MINI, % (*n*)		
PTSD	0 (0)	0 (0)
Depression	0 (0)	0 (0)
Social phobia	0 (0)	0 (0)
Panic disorder	0 (0)	0 (0)
GAD	5 (3)	0 (0)
OCD	0 (0)	0 (0)
On antidepressant medication, % (*n*)	2 (1)	3 (2)
Post-Traumatic Checklist total score, mean (s.d.)	21.5 (5.5)	Not applicable
Depression, Anxiety and Stress Scale 21		
Depression (%; moderate-extreme; 14–28)	2.1 (27)	2.3 (3.6)
Anxiety (%; moderate-extreme; 10–28)	1.5 (2.5)	1.2 (1.6)
Stress (%; moderate-extreme; 19–28)	5.7 (6.1)	3.0 (3.3)*
Dissociative Experiences Scale, mean (s.d.)	4.4 (3.1)	4.0 (3.2)
Connor–Davidson Resilience Scale, mean (s.d.)	77.1 (10.2)	76.9 (12.0)
Post-Traumatic Growth Inventory, mean (s.d.)	48.3 (23.5)	Not applicable
State-Trait Anxiety Inventory		
State	27.8 (7.4)	26.3 (8.1)
Trait	31.1 (7.7)	30.2 (6.9)
Social Adjustment Scale, mean (s.d.)	1.7 (0.3)	1.8 (0.3)
Traumatic Exposure Severity Scale		
Total Occurrence Score (sum of all items)	10.8 (4.2)	Not applicable
Life Events Scale		
Mean number (past 5 years; s.d.)	7.8 (3.8)	6.1 (3.2)*
Mean number (past 6 months; s.d.)	2.5 (2.3)	2.8 (1.7)
Mean difficulty (past 5 years; s.d.)	2.5 (2.3)	1.8 (0.6)*
Childhood Trauma Questionnaire		
Mean rank	51.20	64.68*

MINI, Mini-International Neuropsychiatric Interview; PTSD, post-traumatic stress disorder; GAD, generalised anxiety disorder; OCD, obsessive–compulsive disorder.

*P < 0.05, **P < 0.001.

### Between-group cognitive comparisons

After controlling for demographic and clinical variables (age, gender, years of education, anxiety level from STAI-state, depression symptoms (depression subscale score of the DASS-21), ethnicity, CTQ score and NART scores), the earthquake-exposed group performed significantly better than the non-exposed group on the Total Digit Forward test (F(1,97) = 7.2, *P* = 0.026) (Table 2). No significant between-group differences on any other cognitive measures were found.

**Table 2:** Mean scores on cognitive test variables (non-social measures) in earthquake-exposed and non-exposed groups

	Earthquake-exposed resilient group, *n =* 57, mean (s.e.)	Non-exposed group, *n* = 60, mean (s.e.)	F (d.f.)	*P-*value	Partial eta squared
Verbal learning and memory (RAVLT)					
Number of words					
Trial 1	6.2 (0.6)	7.2 (0.4)	2.3 (1, 97)	0.191	0.20
Total trials 1–5	9.8 (0.6)	10.6 (0.4)	2.1 (1, 97)	0.283	0.15
Distractor list	6.1 (0.6)	6.5 (0.4)	0.0 (1,97)	0.573	0.26
Immediate recall	10.3 (1.0)	10.8 (0.6)	0.7 (1, 97)	0.687	0.12
Delayed recall	9.9 (1.0)	11.0 (0.6)	1.7 (1,97)	0.392	0.14
Visuospatial learning and memory (GMLT)					
Number of errors					
Total trials 1–5	9.0 (0.9)	11.0 (0.6)	3.5 (1, 92)	0.070	0.35
Delayed recall	6.3 (1.4)	8.3 (0.9)	1.9 (1, 92)	0.221	0.28
Executive functioning					
Digit Span Test					
Total digits forward	9.7 (0.7)	7.9 (0.4)	7.2 (1, 97)	0.026*	0.30*
Total digits backward	7.4 (0.7)	7.7 (0.5)	0.0 (1, 97)	0.756	0.26
Forward span	7.3 (0.4)	6.4 (0.2)	3.9 (1, 97)	0.067	0.24
Backward span	5.4 (0.4)	5.2 (0.3)	0.5 (1, 97)	0.778	0.26
Digit forward and backward total	17.1 (1.2)	15.5 (0.8)	2.4 (1, 97)	0.281	0.30
COWAT					0.28
Total	58.8 (5.2)	62.7 (3.3)	0.3 (1, 97)	0.542	
Delis–Kaplan Executive Battery Fluency					0.33
Total words	64.0 (4.1)	60.2 (2.7)	0.4 (1, 97)	0.451	
Delis–Kaplan Executive Battery Switching					
Total words	19.1 (1.3)	16.7 (0.8)	1.1 (1, 97)	0.126	0.29
Total switches	20.5 (1.1)	18.2 (0.7)	2.7 (1, 97)	0.105	0.03
Psychomotor speed					
Timed Chase Test (GMLT)					
Moves per second	1.1 (0.1)	1.5 (0.0)	4.0 (1, 96)	0.055	0.35
Digit Symbol Coding Test (DSCT)					
Total correct	31.7 (2.3)	30.9 (1.5)	0.1 (1, 97)	0.767	0.24
Sustained attention (Vigil CPT)					
Omissions	41.4 (6.1)	45.1 (3.9)	0.2 (1, 95)	0.626	0.004
Commissions	1.9 (1.4)	2.9 (0.9)	0.4 (1, 95)	0.524	0.007
Reaction time (ms)	394 (22)	404 (14)	0.1 (1, 95)	0.724	0.170
Accuracy (%)	97.7 (2.3)	97.4 (1.4)	0.1 (1, 95)	0.917	0.070

Means are estimated means with age, gender, years of education, ethnicity, CTQ and NART scores as covariates. RAVLT, Rey Auditory Verbal Learning Test; GMLT, Groton Maze Learning Test; COWAT, Controlled Oral Word Association Test; DSCT, Digit Symbol Coding Test; Vigil CPT, Vigil Continuous Performance Test.

*P < 0.05.

**Fig. 1:**
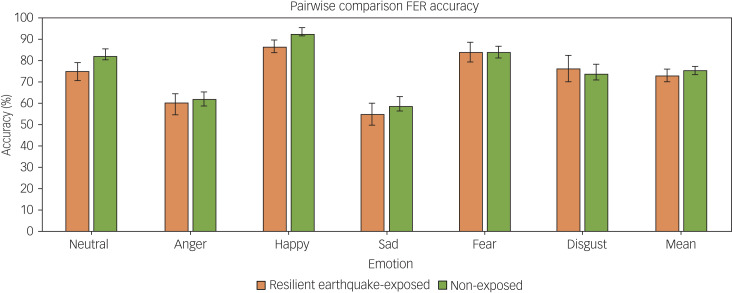
Recognition accuracy (mean and s.e.m.) for the five facial expressions of emotion and neutral expressions on the Facial Expression Recognition Task (FER), in the earthquake-exposed resilient group (*n* = 57) and the non-exposed control group (*n* = 60).

**Fig. 2:**
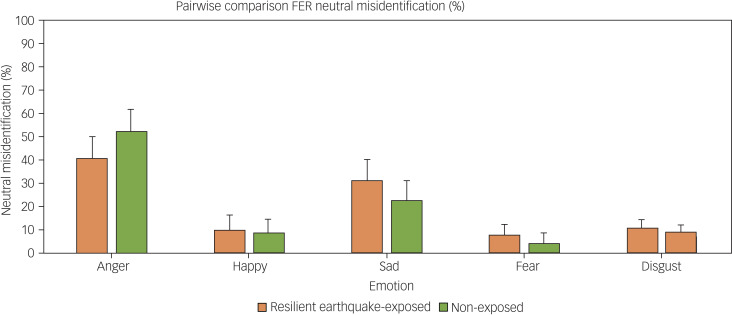
Misclassification (mean and s.e.m.) of neutral expressions to the five facial expressions of emotions on the Facial Expression Recognition Task (FER), in the earthquake-exposed resilient group (*n* = 57) and the non-exposed control group (*n* = 60).

On measures of social cognition, no significant between-group differences were found in total RMET score (F(1,94) = 0.7, *P* = 0.38, partial eta squared 0.23), or any of the FER test variables, including overall reaction time and accuracy, or reaction time or accuracy in identifying specific facial expressions (neutral, angry, happy, sad, fearful and disgusted, all *P* > 0.05, partial eta squared 0.000–0.039; see Fig. 1). No significant between-group differences were found in tendency to misinterpret neutral expressions as particular emotions (see Fig. 2; all *P* > 0.1, partial eta squared 0.002–0.018).

### Within-group cognitive comparisons over time

Of the 57 earthquake-exposed resilient participants in the current study, 50 had completed cognitive testing in the original study 2–3 years after the earthquakes (time point 1), which therefore allowed comparison of these participants over time (see Supplementary Figure 1). Of these 50, one completed FER, but not cognitive testing, meaning 50 participants were included in the FER and 49 in the cognitive analysis. A smaller battery of cognitive tests were completed at time point 1 (RAVLT, GMLT, TCT and DSCT), with comparisons limited to these.

As shown in Table 3, in comparing the earthquake-exposed group at time points 1 and 2, poorer performance on the RAVLT (all variables except distractor list recall) and the TCT, and better performance on one GMLT variable (delay trial, total errors), were found at time point 2 compared with time point 1.

**Table 3:** Means of cognitive test variables in earthquake-exposed resilient group over time

	Earthquake-exposed resilient group time point 1, *n =* 49, mean (s.e.)	Earthquake-exposed resilient group time point 2, *n =* 49, mean (s.e.)	Mean difference (s.e.)	*P*-value	Partial eta-squared
Verbal learning and memory (RAVLT)					
Number of words					
Trial 1	7.8 (0.21)	7.10 (0.29)	−0.71 (0.26)	0.009*	0.147
Total Trial 1−5	11.15 (0.21)	10.44 (0.26)	−0.71 (0.21)	0.001**	0.145
Distractor List	6.74 (0.30)	6.44 (0.19)	−0.30 (0.30)	0.317	0.054
Total Trial 6, immediate recall	11.34 (0.37)	10.23 (0.44)	−1.10 (0.38)	0.006*	0.084
Total Trial 7, delayed recall	11.40 (0.40)	10.18 (0.49)	−1.22 (0.56)	0.010*	0.057
Visuospatial learning and memory (GMLT)					
Number of errors					
Total trials 1−5	10.62 (0.39)	10.01 (0.38)	−0.60 (0.40)	0.140	0.016
Delayed recall	8.85 (0.61)	7.15 (0.47)	1.70 (0.65)	0.013*	0.086
Psychomotor speed					
Timed Chase Test (GMLT)					
Moves per second	1.43 (1.17)	1.31 (0.03)	−0.12 (0.03)	0.000**	0.233
Digit Symbol Coding Test (DSCT)					
Total correct	33.22 (0.94)	33.58 (1.03)	0.36 (0.75)	0.635	0.005

Time point 1: approximately 2–3 years after the beginning of the earthquake sequence; time point 2: approximately 8–9 years after the beginning of the earthquake sequence. RAVLT, Rey Auditory Verbal Learning Test; GMLT, Groton Maze Learning Test; DSCT, Digit Symbol Coding Test.

*P < 0.05, **P < 0.001.

On the FER task, the earthquake-exposed group at time point 2 had slower reaction times for expressions of anger (F(1,42) = 10, *P* = 0.003), sadness (F(1,42) = 9.3, *P* = 0.004) and disgust (F(1,42) = 5.4, *P* = 0.025), compared with time point 1. No differences for the other emotions were found (neutral: F(1,42) = 1.2, P = 0.274; happy: F(1,42) = 1.3, *P* = 0.267; and fearful: F(1,42) = 3.7, *P* = 0.061)). The earthquake-exposed group were also more accurate in identifying neutral expressions (neutral: F(1,42) = 6.1, *P* = 0.018), but not other emotions (angry: F(1,42) = 0.2, *P* = 0.696; happy: F(1,42) = 1.4, *P* = 0.246; sad: F(1,42) = 1.4, *P* = 0.963; fearful: F(1,42) = 0.1, *P* = 0.760; and disgust: F(1,42) = 0.0, *P* = 0.969)) at time point 2 compared with time point 1. No differences in performance across time points were found in neutral misinterpretation scores.

## Discussion

The aim of the current study was to examine the long-term cognitive effects of exposure to the Canterbury earthquake sequence (8–9 years post-earthquake sequence) in a group of people who had previously identified as resilient, by comparing them with a non-exposed control group. Contrary to our findings at 2–3 years post-earthquake, with the exception of a measure of working memory (Digit Span Forward), no significant differences were found in performance between the earthquake-exposed resilient and non-exposed groups in this longer-term comparison.

These findings may be explained by a combination of factors, including the passage of time since the earthquakes and the context in which the studies were conducted. At the time the original studies were conducted, people were living in an environment of ongoing seismic activity where, over a 2-year period, Canterbury experienced over 10 000 aftershocks. This may have resulted in biological changes, such as in the amygdala, with residents being in a chronically hyper-aroused state. At that time, attentional biases to threat, as shown by the facial expression recognition data,^18^ may have been adaptive, with advantage in attending to potential threat rather than underestimating it. By the time of the current study, there was no seismic activity and the sense of threat had abated. Maintaining a constant state of hypervigilance in this context would provide less advantage and may potentially be maladaptive (i.e. playing a role in promoting or maintaining anxiety). In support of this interpretation, facial emotion recognition studies from Italy post-earthquakes reported similar findings, with evidence of hypervigilance in the context of seismic activity,^19,20^ but not in the longer term.^36^ Similarly, a previous study that reported poorer cognitive performance in women with and without PTSD after exposure to intimate partner violence compared with healthy controls, included participants where exposure was relatively recent (between 4 weeks and 2 years previously).^12^ A study four years after an earthquake in Turkey, found impaired performance on a test of immediate verbal recall in those with PTSD, but not in earthquake-exposed controls.^37^ Studies examining even longer-term outcomes, including holocaust survivors with and without PTSD compared with non-exposed controls, reported no difference between non-PTSD and non-exposed participants.^38^ These findings provide early support for the hypothesis that changes in cognitive and emotion processing in trauma-exposed resilient people may be state-dependent, and related to exposure to continued threat in the environment that reverses when threat resolves.

A further explanation for our findings of no difference in cognitive function 8–9 years after the earthquakes between earthquake-exposed resilient and non-exposed groups may relate to the improved design of the current study, which included a concurrently recruited, non-exposed control group. In our earlier studies, the non-exposed comparison was conducted as a supplementary analysis, and included data from historical control groups recruited for two studies conducted before the start of the earthquake sequence. These were therefore not matched to the earthquake-exposed groups, were younger than the non-exposed control group of the current study, and were different from the current non-exposed group in having no past or family history of mental disorder. These historical studies had also utilised slightly different cognitive test batteries so that differences in the length and timing of the batteries between the exposed groups and controls may have resulted in differences in performance.

Further analysis of a subset of the original earthquake-exposed resilient group allowed examination of changes in cognitive functioning over time, comparing time point 1 (approximately 2–3 years after the start of the earthquake sequence) with time point 2 from the current study (approximately 8–9 years after the earthquakes). In the current study, the group performed less well on tests of verbal memory and learning, and were slower on a test of psychomotor speed. These findings may be explained by a deterioration in functioning as a result of ageing of the cohort, with the studies being conducted 6–7 years apart.^39^ Performance on one of the tests of spatial learning and memory, however, was better in the current study (at time point 2) than in the original (at time point 1). Interestingly, in the original study, performance on this test had been impaired compared with the non-exposed control sample. Findings from the FER task included slower reaction times for expressions of emotions of anger, sadness, fear and disgust in the current study compared with the original study. In our original study, we had reported faster responses to anger, fear and disgust (but slower response to sadness) in the earthquake-exposed resilient group compared with the historical non-exposed group. It is possible that improvement in visuospatial performance and slowing of reaction times to negative emotions suggests normalisation of these outcomes over time, when the threat has abated.

These conclusions are, however, speculative, being hampered by the study design, which unfortunately did not allow examination of changes over time in the non-exposed group. Comparisons were also only able to be conducted on 55% of the original sample of earthquake-exposed resilient participants. Although there were very few differences between those who did and did not participate at time point 2, it is possible that those in the current study were more resilient. For example, although there were no differences on measures of resilience, anxiety or depression, those who did not take part in the current study had higher rates of alcohol use, poorer visuospatial performance (*P* = 0.015) and slower reaction times to sad emotions (*P* = 0.015) at time point 1.

Regarding explicit aspects of psychological functioning, the exposed group reported functioning well, had low scores on measures of depression and anxiety, high ratings of resilience and very low rates of mental disorder. This suggests that those identifying as resilient at time point 1, approximately 2–3 years after the earthquakes, maintained this trajectory despite exposure to a significantly higher number of stressful life events and experiencing greater difficulty from these, compared with the non-exposed controls. It is well-established that exposure to adverse childhood experiences increases risk for worse mental health in adulthood,^40^ and it is of note that the earthquake-exposed resilient group had less exposure to childhood trauma than the average population represented by the non-exposed group.

Study findings should be interpreted in the context of the following limitations. Although the groups were well-matched on gender, age and years of education, the earthquake-exposed resilient group scored significantly higher on the NART estimate of premorbid IQ and included a greater proportion of New Zealand European participants. These differences were, however, adjusted for statistically. There was also no measure of affective temperament, which has also been shown to be associated with mental health outcomes.^41^ The earthquake-exposed resilient group comprised 57 of the original 101 participants identified in the studies at time point 1, 2–3 years after the earthquakes.^13,18^ Although the study design was improved by utilising a concurrently recruited, non-exposed control group rather than historical controls, this did not allow for examination of changes in functioning over the 6–7 years between studies in the non-exposed group, which limits the strength of the findings. Although uniformity of earthquake exposure was a strength, it may limit the generalisability of the findings to those exposed to other trauma types in light of previous reports of more prolonged effects after exposure to interpersonal trauma. Results should also be considered in light of the 2 years of extensive seismic activity. This constitutes a particular type of stress, in which there were ongoing aftershocks with potential for them to become serious and life-threatening. About 50% of the non-exposed group were recruited after the start of the COVID-19 pandemic. Although this could have resulted in additional stressors, there was no difference in psychometric measures (DASS-21 scores) between these participants and those recruited before the pandemic (perhaps reflecting the positive COVID-19 response in New Zealand at the time). Subanalyses of cognitive outcomes were not possible because of the size of the groups. A final issue is that of limited statistical power related to the size of the groups, which may have resulted in type 2 errors.

The study has considerable strengths related to the study design. These include all of the earthquake-exposed participants having been exposed to the same type of trauma at the same time, whereas most other studies have included participants exposed to a wide variety of types of trauma. All exposed participants were resilient and therefore did not have comorbidities such as depression, which complicate interpretation of cognitive findings in many previous studies.

In conclusion, the key finding from the current study was that no differences were found in performance on any of the cognitive tasks in individuals who had been exposed to earthquakes 8–9 years previously but identified as resilient, compared with matched non-exposed controls. There are some limitations in the strength of these findings related to study design and because we were not able to robustly examine changes in cognitive functioning over time. However, if replicated, they suggest that changes in cognitive functioning in trauma-exposed resilient people may be state-dependent and related to exposure to continued threat in the environment, which improves as the threat reduces. The findings increase our understanding of the effects of trauma exposure in resilient populations, and highlight the importance of examining longer-term outcomes.

## Data Availability

The data that support the findings of this study are available from the corresponding author, C.B., upon reasonable request.

## Supplementary material

For supplementary material accompanying this paper visit http://doi.org/10.1192/bjo.2022.512. click here to view supplementary material

## References

[1] Ardagh MW, Richardson SK, Robinson V, Than M, Gee P, Henderson S, The initial health-system response to the earthquake in Christchurch, New Zealand. Lancet 2012; 379(9831): 2109–15. 22510397 10.1016/S0140-6736(12)60313-4

[2] Benjet C, Bromet E, Karam EG, Kessler RC, McLaughlin KA, Ruscio AM, The epidemiology of traumatic event exposure worldwide: results from the World Mental Health Survey Consortium. Psychol Med 2016; 46(2): 327–43. 26511595 10.1017/S0033291715001981 PMC4869975

[3] Beaglehole B, Mulder RT, Frampton CM, Boden JM, Newton-Howes G, Bell CJ. Psychological distress and psychiatric disorder after natural disasters: systematic review and meta-analysis. Br J Psychiatry 2018; 213(6): 716–22. 30301477 10.1192/bjp.2018.210

[4] Fergusson DM, Horwood LJ, Boden JM, Mulder RT. Impact of a major disaster on the mental health of a well-studied cohort. JAMA Psychiatry 2014; 71(9): 1025–31. 25028897 10.1001/jamapsychiatry.2014.652

[5] Galatzer-Levy IR, Huang SH, Bonanno GA. Trajectories of resilience and dysfunction following potential trauma: a review and statistical evaluation. Clin Psychol Rev 2018; 63: 41–55. 29902711 10.1016/j.cpr.2018.05.008

[6] Stark EA, Parsons CE, Van Hartevelt TJ, Charquero-Ballester M, McManners H, Ehlers A, Post-traumatic stress influences the brain even in the absence of symptoms: a systematic, quantitative meta-analysis of neuroimaging studies. Neurosci Biobehav Rev 2015; 56: 207–21. 26192104 10.1016/j.neubiorev.2015.07.007

[7] Shin LM, Liberzon I. The neurocircuitry of fear, stress, and anxiety disorders. Neuropsychopharmacol 2010; 35(1): 169–91. 10.1038/npp.2009.83 PMC3055419 19625997

[8] Li Y, Hou X, Wei D, Du X, Zhang Q, Liu G, Long-term effects of acute stress on the prefrontal-limbic system in the healthy adult. PLoS One 2017; 12(1): e0168315. 28045980 10.1371/journal.pone.0168315 PMC5207406

[9] Brewin CR, Kleiner JS, Vasterling JJ, Field AP. Memory for emotionally neutral information in posttraumatic stress disorder: a meta-analytic investigation. J Abnorm Psychol 2017; 116(3): 448–63. 10.1037/0021-843X.116.3.448 17696700

[10] Johnsen GE, Asbjørnsen AE. Consistent impaired verbal memory in PTSD: a meta-analysis. J Affect Disord 2008; 111(1): 74–82. 18377999 10.1016/j.jad.2008.02.007

[11] Crowe M. ‘Quake brain': coping with the series of earthquakes in Christchurch. Int J Ment Health Nurs 2011; 20(6): 381–2. 22236247 10.1111/j.1447-0349.2011.00772.x

[12] Stein MB, Kennedy CM, Twamley EW. Neuropsychological function in female victims of intimate partner violence with and without posttraumatic stress disorder. Biol Psychiatry 2002; 52(11): 1079–88. 12460691 10.1016/s0006-3223(02)01414-2

[13] Bell CJ, Frampton CM, Colhoun HC, Douglas KM, McIntosh VV, Carter FA, Earthquake brain: Impairment of spatial memory following long-term earthquake-related stress. Aust N J Psychiatry 2019; 53(1): 37–47. 10.1177/0004867418789498 30052053

[14] Flaks MK, Malta SM, Almeida PP, Bueno OF, Pupo MC, Andreoli SB, Attentional and executive functions are differentially affected by post-traumatic stress disorder and trauma. J Psychiat Res 2014; 48(1): 32–9. 24199652 10.1016/j.jpsychires.2013.10.009

[15] Couette M, Mouchabac S, Bourla A, Nuss P, Ferreri F. Social cognition in post-traumatic stress disorder: a systematic review. Br J Clin Psychol 2020; 59(2): 117–38. 31696974 10.1111/bjc.12238

[16] MacNamara A, Post D, Kennedy AE, Rabinak CA, Phan KL. Electrocortical processing of social signals of threat in combat-related post-traumatic stress disorder. Biol Psychol 2013; 94(2): 441–9. 24025760 10.1016/j.biopsycho.2013.08.009

[17] Poljac E, Montagne B, de Haan EH. Reduced recognition of fear and sadness in post-traumatic stress disorder. Cortex 2011; 47(8): 974–80. 21075363 10.1016/j.cortex.2010.10.002

[18] Bell CJ, Colhoun HC, Frampton CM, Douglas KM, McIntosh VVW, Carter FA, Earthquake brain: altered recognition and misclassification of facial expressions are related to trauma exposure but not posttraumatic stress disorder. Front Psychiatry 2017; 8: 278. 29312012 10.3389/fpsyt.2017.00278 PMC5732911

[19] Pistoia F, Conson M, Carolei A, Dema MG, Splendiani A, Curcio G, Post-earthquake distress and development of emotional expertise in young adults. Front Behav Neurosci 2018; 12: 91. 29867392 10.3389/fnbeh.2018.00091 PMC5951935

[20] Pistoia F, Conson M, Quarantelli M, Panebianco L, Carolei A, Curcio G, Neural correlates of facial expression recognition in earthquake witnesses. Front Neurosci 2019; 13: 1038. 31611769 10.3389/fnins.2019.01038 PMC6776974

[21] Schmidt M. Rey auditory verbal learning test: a handbook. Western Psychological Services, 1996.

[22] Delis DC, Kaplan E, Kramer JH. Delis-Kaplan Executive Function System (D-KEFS). The Psychological Corporation, 2001.

[23] Cegalis J, Bowlin J. VIGIL: Software for the Assessment of Attention. Forthought, 1991.

[24] Baron-Cohen S, Wheelwright S, Hill J, Raste Y, Plumb I. The “Reading the Mind in the Eyes” test revised version: a study with normal adults, and adults with Asperger syndrome or high-functioning autism. J Child Psychol Psyc 2001; 42(2): 241–51. 11280420

[25] Harmer CJ, Bhagwagar Z, Perrett DI, Völlm BA, Cowen PJ, Goodwino GM. Acute SSRI administration affects the processing of social cues in healthy volunteers. Neuropsychopharmacol 2003; 28(1): 148–52. 10.1038/sj.npp.1300004 12496951

[26] Douglas KM, Porter RJ. Recognition of disgusted facial expressions in severe depression. Br J Psychiatry 2010; 197(2): 156–7. 20679270 10.1192/bjp.bp.110.078113

[27] Weathers FW, Litz BT, Herman DS, Huska JA, Keane TM. The PTSD Checklist (PCL): reliablity, validity, and diagnostic utility. *9th Annual Conference of the ISTSS* (San Antonio, USA). International Society for Traumatic Stress Studies, 1993.

[28] Ng F, Trauer T, Dodd S, Callaly T, Campbell S, Berk M. The validity of the 21-item version of the Depression Anxiety Stress Scales as a routine clinical outcome measure. Acta Neuropsychiatrica 2007; 19(5): 304–310. 26952943 10.1111/j.1601-5215.2007.00217.x

[29] Bernstein EM, Putnam FW. Development, reliability, and validity of a dissociation scale. J Nerv Ment Dis 1986; 174(12): 727–35. 3783140 10.1097/00005053-198612000-00004

[30] Tedeschi RG, Calhoun LG. The Posttraumatic Growth Inventory: measuring the positive legacy of trauma. J Trauma Stress 1996; 9(3): 455–71. 8827649 10.1007/BF02103658

[31] Connor KM, Davidson JR. Development of a new resilience scale: the Connor-Davidson Resilience Scale (CD-RISC). Depress Anxiety 2003; 18(2): 76–82. 12964174 10.1002/da.10113

[32] Weissman MM, Bothwell S. Assessment of social adjustment by patient self-report. Arch Gen Psychiatry 1976; 33(9): 1111–5. 962494 10.1001/archpsyc.1976.01770090101010

[33] Elal G, Slade P. Traumatic Exposure Severity Scale (TESS): a measure of exposure to major disasters. J Trauma Stress 2005; 18(3): 213–20. 16281215 10.1002/jts.20030

[34] Shalowitz MU, Berry CA, Rasinski KA, Dannhausen-Brun CA. A new measure of contemporary life stress: development, validation, and reliability of the CRISYS. Health Serv Res 1998; 33(5 Pt 1): 1381–402. 9865225 PMC1070321

[35] Bernstein DP, Fink L, Handelsman L, Foote J, Lovejoy M, Wenzel K, Initial reliability and validity of a new retrospective measure of child abuse and neglect. Am J Psychiatry 1994; 151(8): 1132–6. 8037246 10.1176/ajp.151.8.1132

[36] Sagliano L, Conson M, Saporito G, Carolei A, Sacco S, Pistoia F. “Far from the mind”: preliminary evidence of avoidance bias for emotional facial expressions among earthquake victims. Int J Disast Risk Reduct 2021; 59: 102273.

[37] Eren-Koçak E, Kiliç C, Aydin I, Hizli FG. Memory and prefrontal functions in earthquake survivors: differences between current and past post-traumatic stress disorder patients. Acta Psychiat Scand 2009; 119(1): 35–44. 18853946 10.1111/j.1600-0447.2008.01281.x

[38] Golier JA, Yehuda R, De Santi S, Segal S, Dolan S, de Leon MJ. Absence of hippocampal volume differences in survivors of the Nazi Holocaust with and without posttraumatic stress disorder. Psychiatry Res 2005; 139(1): 53–64. 15939577 10.1016/j.pscychresns.2005.02.007

[39] Gray V, Douglas KM, Porter RJ. Emotion processing in depression and anxiety disorders in older adults: systematic review. BJPsych Open 2020; 7(1): e7. 33267933 10.1192/bjo.2020.143 PMC7791559

[40] Hughes K, Bellis MA, Hardcastle KA, Sethi D, Butchart A, Mikton C, The effect of multiple adverse childhood experiences on health: a systematic review and meta-analysis. Lancet Public Health 2017; 2(8): e356–66. 29253477 10.1016/S2468-2667(17)30118-4

[41] Baldessarini RJ, Innamorati M, Erbuto D, Serafini G, Fiorillo A, Amore M, Differential associations of affective temperaments and diagnosis of major affective disorders with suicidal behavior. J Affect Disord 2017; 210: 19–21. 27992854 10.1016/j.jad.2016.12.003

