# Supplementary material for: Examining the long-term cognitive effects of exposure to the Canterbury earthquakes in a resilient cohort
Source: BJPsych Open. 2022 Jun 15;8(4):e114. doi: 10.1192/bjo.2022.512 (PMC9230545; doi:10.1192/bjo.2022.512)
Supplement: Supplementary file 1 [file S2056472422005129sup001.docx]

**CURRENT STUDY**

Within-group comparison over time

101 Earthquake-exposed resilient participants recruited at Time 1

57 Earthquake-exposed resilient participants cognitive testing at Time 2 compared with 60 non-exposed controls

**ORIGINAL STUDY**

50 of these 57 participants completed testing at Time 1 and Time 2

89 Earthquake-exposed resilient participants cognitive testing at Time 1 compared with 28 PTSD and historical non-exposed controls

59 Earthquake-exposed participants recruited at Time 2

42 participants not tested at Time 2

**CURRENT STUDY**

Between-group comparison
